# Muscle–Organ Crosstalk: The Emerging Roles of Myokines

**DOI:** 10.1210/endrev/bnaa016

**Published:** 2020-05-11

**Authors:** Mai Charlotte Krogh Severinsen, Bente Klarlund Pedersen

**Affiliations:** Centre of Inflammation and Metabolism/Centre for Physical Activity Research (CIM/CFAS), Rigshospitalet, University of Copenhagen, Copenhagen, Denmark

**Keywords:** metabolism, cytokines, exercise, physical activity, diabetes, cancer

## Abstract

Physical activity decreases the risk of a network of diseases, and exercise may be prescribed as medicine for lifestyle-related disorders such as type 2 diabetes, dementia, cardiovascular diseases, and cancer. During the past couple of decades, it has been apparent that skeletal muscle works as an endocrine organ, which can produce and secrete hundreds of myokines that exert their effects in either autocrine, paracrine, or endocrine manners. Recent advances show that skeletal muscle produces myokines in response to exercise, which allow for crosstalk between the muscle and other organs, including brain, adipose tissue, bone, liver, gut, pancreas, vascular bed, and skin, as well as communication within the muscle itself. Although only few myokines have been allocated to a specific function in humans, it has been identified that the biological roles of myokines include effects on, for example, cognition, lipid and glucose metabolism, browning of white fat, bone formation, endothelial cell function, hypertrophy, skin structure, and tumor growth. This suggests that myokines may be useful biomarkers for monitoring exercise prescription for people with, for example, cancer, diabetes, or neurodegenerative diseases.

## Graphical Abstract

**Graphical Abstract:**
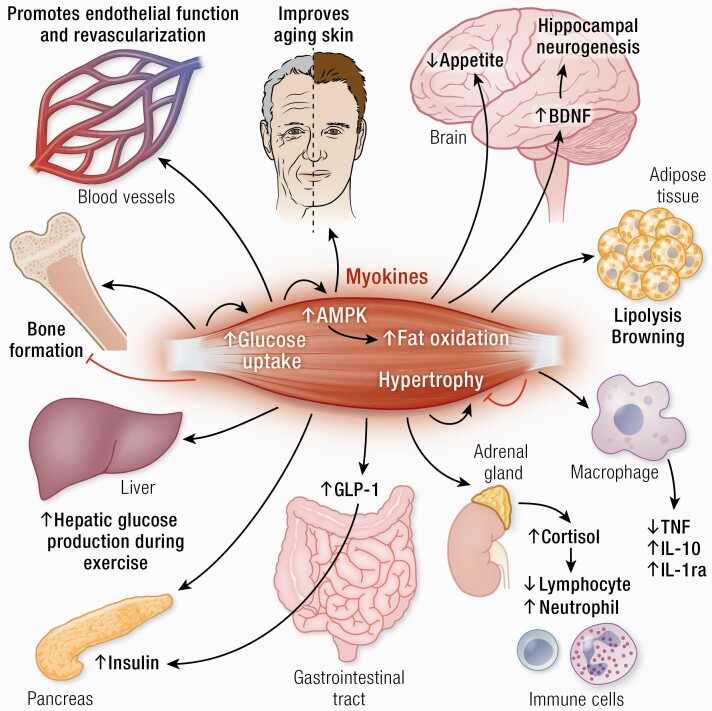

**Essential Points**

- Myokines are defined as cytokines and other peptides that are produced, expressed and released by muscle fibers and exert either autocrine, paracrine, or endocrine effects
- Myokines mediate communication between muscle and other organs, including brain, adipose tissue, bone, liver, gut, pancreas, vascular bed, and skin, as well as within the muscle itself
- Myokines exert their effects on, for example, cognition, lipid and glucose metabolism, browning of white fat, bone formation, endothelial cell function, hypertrophy, skin structure, and tumor growth
- The myokine IL-6 mediates the exercise-associated anti-inflammatory effects both acutely with each bout of exercise and as a consequence of training adaptation, including reduction in abdominal adiposity.
- The identification of new myokines and their specific roles may lead to novel therapeutic targets
- Myokines can be useful biomarkers for monitoring the type and amount of exercise that are required for the prescription of exercise for people with, for example, cancer, diabetes, or neurodegenerative diseases

Within the society of human integrative physiology, the awareness of an exercise factor that is able to mediate exercise-induced changes in other organs such as liver and adipose tissue dates back more than 50 years. It was clear that signaling pathways from exercising skeletal muscle to other organs were not solely mediated via the nervous system, since electrical stimulation of paralyzed muscles in patients with no efferent or afferent nerve impulses induced the same types of physiological changes as were found in healthy human beings (1, 2). Thus, it was obvious that 1 or several humoral factors had to be released from contracting muscles to the blood (3).

Before such factors were identified, they were referred to as the “work factor” or the “exercise factor” (4). Our finding in 2000 that skeletal muscle produced and released interleukin-6 (IL-6) into the circulation (5) as well as research during the subsequent years, demonstrating that IL-6 has multiple metabolic effects in other parts of the body (6), identified IL-6 as an exercise factor and skeletal muscle as a secretory organ with endocrine functions.

Given the multiple physiological, metabolic, and immunological effects of exercise, it was obvious that more than 1 exercise factor was likely to be found. In 2003, we introduced the term “myokines” (4) and suggested that “cytokines and other peptides that are produced, expressed and released by muscle fibers and exert either autocrine, paracrine or endocrine effects should be classified as myokines” (4, 7).

Following the identification of muscle-derived IL-6, it soon became clear that muscles were able to secrete hundreds of peptides. Although the biological function has been described for only 5% of all known myokines, the identification of the myokinome has provided a new paradigm and a conceptual basis for understanding by which mechanisms muscles communicate with other organs. It has been proposed that the total sum of all exercise-induced factors (such as peptides and nucleic acids) released from muscle and other organs into the blood should be named “exerkines” (8, 9). Exerkines may be released within extracellular vesicles known as exosomes (10), which may contain nucleic acids, peptides, messenger ribonucleic acid (mRNA), microRNA and mitochondrial deoxyribonucleic acid. Although there is an overlap between myokines and exerkines, the present review focuses on myokines.

The role of myokines has previously been reviewed (7, 11-37), identifying more than 650 myokines (38). Some myokines are responsible for mediating energy supply in relation to acute bouts of exercise. Myokines are also involved in muscle proliferation, differentiation, and regeneration independent of exercise (39, 40). During exercise, myokines signal within the muscle and mediate muscle–organ crosstalk to the brain, adipose tissue, bone, liver, gut, pancreas, vascular bed, and skin (7, 29, 30). In addition, myokines with anticancer effects have been recognized (41, 42). The aim of the present review is to provide an update of recent advances within the myokine field.

## Muscle–Muscle Crosstalk

### Myogenesis

**Figure 1.**
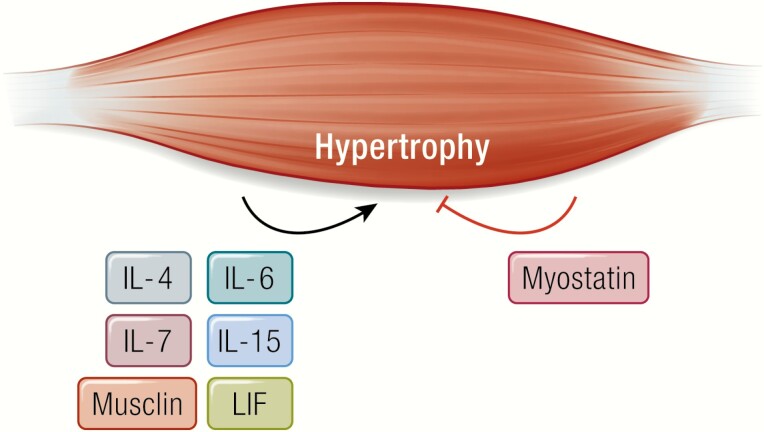
Musclin, LIF, IL-4, IL-6, IL-7, and IL-15 promote muscle hypertrophy. Myostatin inhibits muscle hypertrophy.

Some myokines exert their effect within skeletal muscle itself and are involved in the regulation of muscle mass (14) (Fig. 1).

Myostatin was the first identified muscle-derived factor that fulfills the myokine criteria as outlined above (43). Myostatin is a member of the transforming growth factor β (TGF-β) superfamily and negatively regulates myogenesis in an autocrine manner (43). Massive muscle hypertrophy is seen in myostatin knockout mice, cattle, sheep, and dogs (43-45) that demonstrate an increase in fiber cross-sectional area and in fiber number.

Decorin has been identified as a myokine that is regulated by exercise and acts as an antagonist to myostatin (46). Circulating levels of decorin are increased in response to exercise in humans (46), whereas exercise training reduces the levels of myostatin within muscles and blood (47, 48).

Although the myokine IL-6 is mostly recognized for its regulatory effects in lipid and glucose metabolism, IL-6 also plays important roles in myogenesis. Muñoz-Cánoves and her team identified IL-6 as an anabolic factor in preclinical models. Genetic loss of IL-6 impaired muscle hypertrophy in vivo, whereas myotube-produced IL-6 stimulated muscle cell proliferation in a paracrine fashion (49).

Leukemia inhibitory factor (LIF) is a member of the IL‑6 cytokine superfamily and has multiple biological functions. LIF protein has been shown to be secreted from human cultured myotubes; when electrically stimulated (50) LIF stimulates satellite cell proliferation (51). It has further been shown that both IL-6 and LIF activate myotube mTORC1 signaling in a time- and dose-dependent fashion (52).

A number of other myokines, including IL‑15 (53) and IL-7 (54) have further been demonstrated to possess anabolic features in rodent models.

### Metabolic actions

While IL-6 is characterized as a myokine with endocrine effects, it also works in a paracrine manner exerting metabolic effects within the muscle itself (6, 7).

Physical inactivity is associated with high circulating basal levels of IL-6 in humans (55). Moreover, the acute exercise-induced rise in systemic levels of IL-6 and muscular IL-6 mRNA are diminished by training in humans (56). In contrast, the muscular expression of the IL-6 receptor (IL-6R) is elevated in trained human muscle (57), suggesting that muscular sensitivity to IL-6 is increased by training adaptation. IL-6 signaling within the muscle can affect both glucose uptake and fat oxidation.

It is well documented that IL-6 increases both basal glucose uptake and glucose transporter GLUT4 translocation (58). In addition, IL-6 increases insulin-stimulated glucose uptake in vitro and in healthy humans in vivo. Thus, when recombinant human IL-6 (rhIL-6) was infused into healthy humans together with a hyperinsulinemic, euglycemic clamp, it improved peripheral insulin-stimulated glucose uptake. The effects of IL-6 on glucose uptake in vitro was shown to be mediated by activation of adenosine 5′-monophosphate-activated protein kinase (AMPK) (58). Several other studies have described that IL-6 can increase intramyocellular (58-60) or whole body (61) fatty acid oxidation via AMPK activation (58, 62).

Brain-derived neurotrophic factor (BDNF) is also expressed in human skeletal muscles, but BDNF is not released into the circulation and does not work in an endocrine way. In contrast, BDNF is identified as a myokine capable of enhancing AMPK activation and hence lipid oxidation in an autocrine or paracrine manner (63).

Musclin has been identified as an exercise-induced factor (64) promoting skeletal muscle mitochondrial biogenesis in mice (65). Recent evidence shows that musclin abolishes muscle atrophy related with cancer in mice (66).

## Muscle–Brain Crosstalk

Evidence is accumulating that physical exercise has positive health effects on cognitive function and brain health (67, 68). Physical activity and exercise training decrease the risk of dementia (69-71) and appear to play a role in the treatment of this disease (72). In general, it is found that physical activity decreases the rate of cognitive decline in healthy people and in people with neurodegenerative disorders across the life span (73). Moreover, physical exercise has a positive impact on stress, anxiety, and depression (72). Other studies have shown that an active lifestyle is associated with learning and memory (74), executive functions (75), language and reaction time (76), academic achievement in children, and intelligence in adolescents (77). Physical activity has also beneficial effects on appetite (78), sleep (79), and mood (80).

Exercise has been shown to influence the hippocampus more than any other part of the brain. Studies in rodents (81) and humans (82) have shown that exercise increases hippocampus volume and the blood flow to this part of the brain (81). In particular, exercise has been shown to influence neurogenesis in the dentate gyrus (67, 68) and to increase synapse plasticity (67, 68).

**Figure 2.**
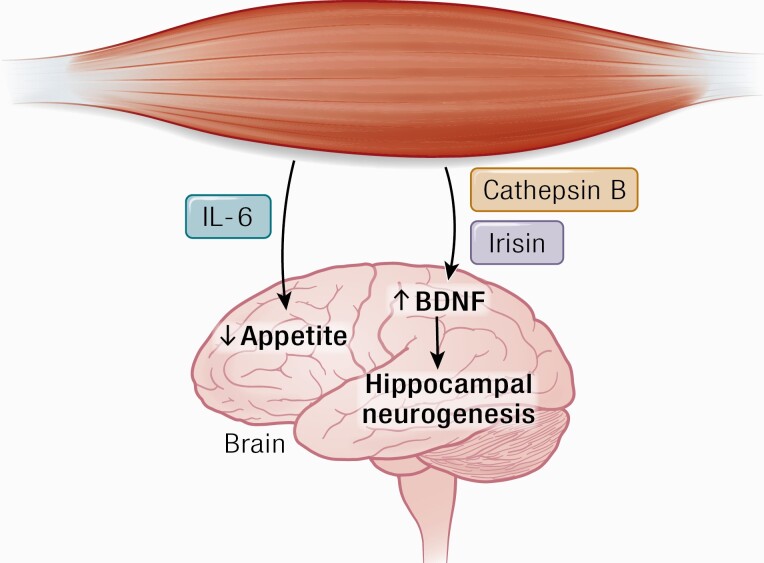
Cathepsin B and irisin cross the blood–brain barrier and stimulate BDNF production, which leads to hippocampal neurogenesis. IL-6 stimulates appetite. Abbreviations: BDNF, brain-derived neurotrophic factor.

The finding that muscle contraction is sensed by the brain suggests that peripheral factors induced by exercise may be involved in direct crosstalk between working muscle and brain function (7, 29, 30, 83) (Fig. 2).

### Cognition, hippocampal neurogenesis, and learning

Recent findings suggest that a muscle–brain endocrine loop exists, which at least in part may be mediated by myokine signaling. Other possible mediators include various metabolites (84), noncoding RNAs (85), hormonal responses, and muscular enzymes with impact on circulating compounds (30). BDNF appears to play a dominant role in mediating the effects of exercise on hippocampus (86). Rodent studies demonstrate increased BDNF mRNA and BDNF protein within the hippocampus in response to wheel running for 1-8 weeks (87-92). Furthermore, BDNF has been shown to be mechanistically linked to exercise-induced improvement in cognitive functions, such as memory and learning (93, 94).

Studies in humans show that BDNF is released from the brain during a bout of bicycle exercise (95, 96), and aerobic exercise training for 3 months increases the volume of the hippocampus in healthy individuals by 12% and by 16% in patients with schizophrenia (97). BDNF is a growth factor for the hippocampus and involved in, for example, cell survival and learning (98). The finding that BDNF is also expressed in human skeletal muscle during exercise is interesting; however, muscle-derived BDNF has not been shown to be released from muscle into the blood stream, thereby mediating a direct muscle–brain interaction (63).

A couple of interesting studies propose that the myokines cathepsin-B and irisin may pass the blood–brain barrier and provoke an increase in BDNF. Moon et al. (99) recently identified a novel myokine, cathepsin B (CTSB) (99) and demonstrated in a series of elegant studies that exercise leads to elevated systemic levels of CTSB, which promote expression of BDNF in the hippocampus and stimulate neurogenesis. Running led to an increased muscular expression of the *CTSB* gene in mice and an increase in CTSB in plasma from mice, rhesus monkeys, and in humans following treadmill running for 4 months. CTSB was furthermore shown to pass the blood–brain barrier in mice. Moon et al. (99) also performed studies in CTSB knockout mice and showed that mice lacking CTSB were resistant to an effect of voluntary exercise as regards hippocampal growth and improved cognition. It is not known if the myokine CTSB mediates enhanced cognitive functions in humans in response to exercise training (99, 100).

The PGC-1α-dependent myokine irisin, known for its browning effects (101), may also be involved in mediating effects of physical activity on the brain (98). When irisin is overexpressed in primary cortical neurons, it leads to an increase in BDNF expression, whereas RNAi-mediated knockdown of FNDC5 is followed by a reduction of BDNF. Moreover, systemic levels of irisin is elevated when irisin is delivered to the murine liver via adenoviral vectors, which leads to increased levels of BDNF in the hippocampus. It is controversial whether exercise raises plasma concentrations of irisin in humans (102, 103), and whether irisin is involved in a muscle–brain endocrine loop.

### Appetite

Elevated levels of IL-6 accompany, for example, obesity and type 2 diabetes (7), and IL-6 is often linked with the metabolic syndrome, not least in animal models (104, 105). However, IL-6 has also been shown to affect metabolic actions beneficially. IL-6-deficient mice gain weight and develop whole-body insulin resistance (106, 107). Other rodent studies show that IL-6 triggers proliferation of pancreatic alpha cells in the obese state (108) and stimulates the production of glucagon-like peptide (GLP)-1 and hence insulin secretion (108). Studies in murine macrophages and hepatocytes show that IL-6 improves glucose homeostasis (109, 110).

Human studies demonstrate that physiological levels of IL-6 have many positive effects, including an enhancement of both insulin-stimulated glucose uptake (58) and lipolysis and fat oxidation (61). IL-6 also delays gastric emptying and thereby exerts effects on postprandial glucose control (111). Infusion of IL-6 to humans stimulates the production of IL-1ra and IL-10 (112) and inhibits endotoxin-induced tumor necrosis factor (TNF) production (113), thereby inducing anti-inflammatory effects.

During muscle work, IL-6 is produced by human contracting skeletal muscle and released into the blood (114) in a TNF-independent fashion (115). The release of IL-6 leads to an exponential rise in circulating concentrations of IL-6 in humans. Systemic IL-6 knockout mice accumulate adipose tissue (106, 107), whereas central overexpression of IL-6 (116, 117) leads to a decrease in body weight, indicating that IL-6 is a player in body weight control. Another murine study demonstrated that lack of muscular IL-6 led to a decrease in body weight and food consumption in response to leptin (118).

A study showed that IL-6 improves glucose tolerance and suppresses feeding when it is applied centrally in mice, but not intraperitoneally at the same dose (119). However, a 4-fold higher IL-6 concentration injected peripherally significantly reduced food intake. This finding suggests that high systemic concentrations of IL-6 can pass the blood–brain barrier and exert central effects on appetite. Thus, it is likely that muscle-derived IL-6, elicited by exercise of long duration and high intensity, may inhibit appetite.

## Muscle–Adipose Crosstalk

**Figure 3.**
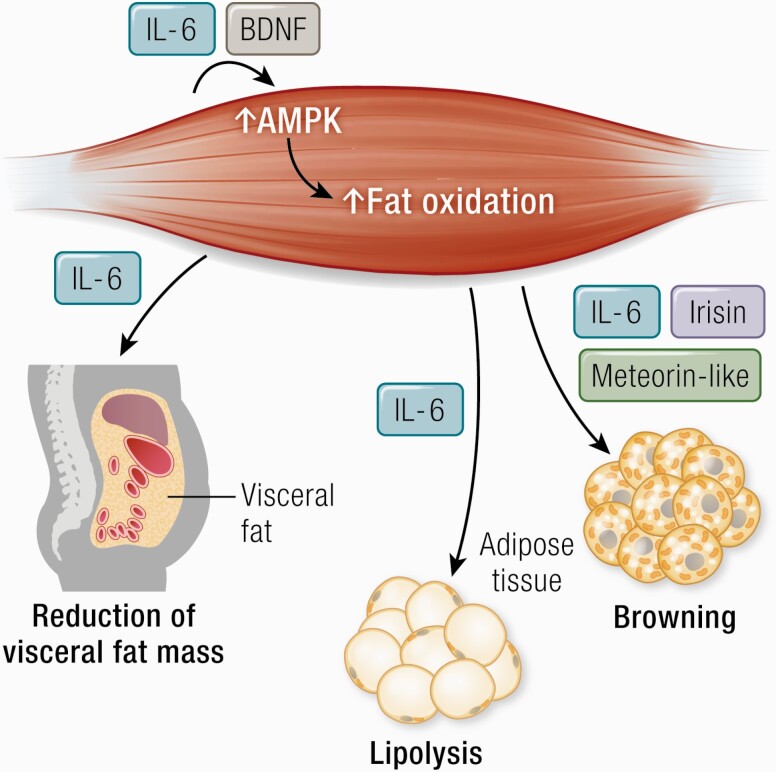
IL-6 stimulates lipolysis decreases visceral fat mass. Irisin, meteorin-like, and IL-6 have a role in “browning” of white adipose tissue. IL-6 and BDNF stimulate AMPK activation. Abbreviations: AMPK, 5′-AMP-activated protein kinase; BDNF, brain-derived neurotrophic factor.

Myokines are involved in the regulation of lipid metabolism in relation to exercise and recent evidence suggests that some myokines may also have the capacity to induce browning of white adipose tissue (Fig. 3).

### Lipolysis

The effect of exercise-induced IL-6 on fat metabolism is one of the most well supported findings (120, 121). In vitro studies and studies in rodents show that IL-6 can enhance lipolysis and fat oxidation, via a mechanism that involves AMPK activation (6). In vivo studies show that rhIL-6 enhances lipolysis and fat oxidation in healthy young and elderly humans (60, 61) and IL-6 autoantibodies appear to be involved in the pathogenesis of a subset of type 2 diabetes (122).

Abdominal adiposity is associated with type 2 diabetes (123), cardiovascular disease (124), dementia (125), colon cancer (126), and breast cancer (127). Abdominal adiposity is also associated with all‐cause mortality, both in obese people and in people with a normal body weight (128). Epidemiological studies clearly show that an association exists between abdominal adiposity and low fitness (129, 130) as well as between abdominal adiposity and low-grade inflammation (129-132). Intervention studies show that physical inactivity promotes an increase in the amount of visceral adipose tissue (29, 133), whereas exercise training diminishes visceral adipose tissue mass (134, 135).

It was, however, not until recently that a mechanism underlying the link between exercise and abdominal fat was established (136). Abdominally obese humans were randomized to tocilizumab (IL-6 receptor antibody) or placebo during an intervention of 12 weeks with either aerobic exercise or no exercise (136, 137). As expected, exercise training led to a reduction in visceral adipose tissue mass. However, this effect was abolished by IL-6 receptor blockade (136). Moreover, IL-6 receptor blockade abolished the exercise-induced loss of cardiac fat (138).

### Browning

Brown fat expresses a set of proteins, such as uncoupling protein 1 (UCP1). The fact that white adipose tissue can shift into a brown-like phenotype, the discovery of brown fat in humans, and the potentially beneficial effects of these depots have stimulated a number of studies to explore whether lifestyle, such as exercise, can contribute to induce browning of white fat (12, 17, 139).

In 2012, irisin was reported as a myokine with the ability to brown white adipose tissue in mice. It was shown that muscular PGC1-α expression stimulates an increase in the expression of the membrane FNDC5 that is cleaved and secreted as irisin. Cell culture studies demonstrated that irisin stimulates UCP1 expression and other brown fat-like genes (101). However, while evidence exists that irisin is released from rodent muscle and has browning effects, it is debated whether exercise leads to an increase in plasma irisin levels in humans. The controversy is mainly based on the fact that previous studies have used commercial enzyme-linked immunosorbent assay kits for irisin, which seems to be unspecific (102, 140).

A couple of other exercise-induced myokines with browning effects have been identified. In 2014, Spiegelman’s group (141) identified meteorin-like (Metrnl), a circulating muscle-derived factor, that is induced in muscle after exercise. Metrnl stimulates the expression of genes associated with beige fat thermogenesis, it further stimulates energy expenditure and improves glucose tolerance. Yet, the role of Metrnl in humans remains to be identified.

A world of literature has proven that IL-6 is released from contracting human muscle cells into the circulation and that it contributes to the exponential increase in plasma IL-6 in relation to exercise, reviewed in 29, 120, 142-145. Studies suggest that IL-6 can induce browning of white adipose tissue. Daily intraperitoneal injections of IL-6 to mice for 1 week increased UCP1 mRNA in inguinal white adipose tissue (146).

A study by Kristóf et al. (147) found that IL-6 was mainly produced by fully differentiated adipocytes. When the IL-6 receptor was blocked during differentiation, brown marker genes were downregulated, suggesting that beige adipocytes regulate IL-6 production to enhance browning in an autocrine manner. It remains to be shown that the physiological concentrations of IL-6, released during exercise, have browning effects.

There are a few other circulating factors during exercise, which have the potential to induce browning. β-Aminoisobutyric acid is a small molecule, a nonprotein beta-amino acid, not classified as a myokine, but secreted from myocytes (148, 149). Moreover, β-aminoisobutyric acid has browning effects on human adipocytes (148, 149). In addition, 2 hepatokines appear to play a role in exercise-induced browning of white adipose tissue. The Fibroblast growth factor 21 (FGF-21) (150) and Follistatin (151) are released from human liver during exercise and this release is controlled by the glucagon-to-insulin ratio (152). Evidence exists that both Follistatin (153) and FGF-21 (154) can induce browning of white adipose tissue cells.

The finding that circulating factors during exercise may induce browning of white adipose tissue has so far largely been restricted to rodents and has not been consistently demonstrated in humans (155, 156).

## Muscle–Bone Crosstalk

**Figure 4.**
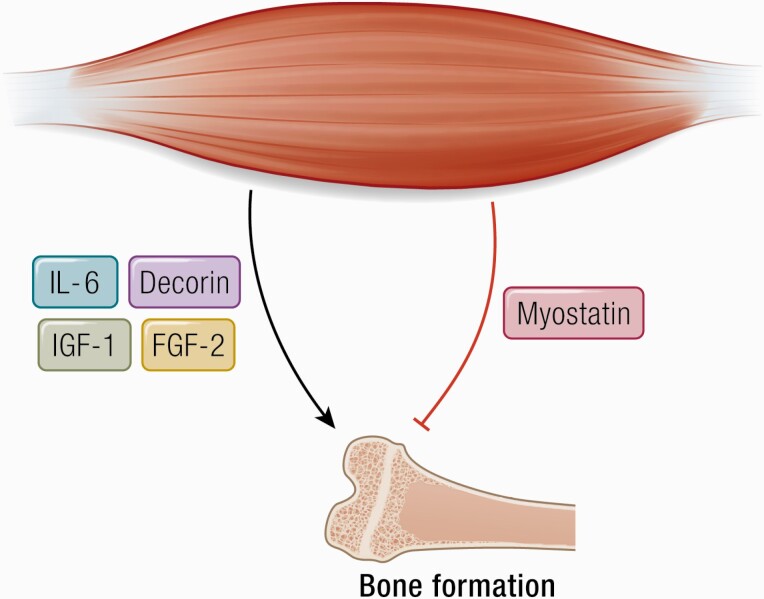
Decorin, IL-6, IGF-1, and FGF-2 positively regulate bone formation. Abbreviations: FGF-2, fibroblast growth factor 2; IGF-1, insulin-like growth factor I.

Muscle and bone are closely related during development growth (157), and muscle disuse and/or muscle atrophy result in osteoporosis (158). As pointed out by Guo et al. (159), muscle mass, measured as lean body mass, can only explain up to 20% of the variety in bone mineral density (158) and decreased mechanical loading, as seen with muscle atrophy alone, is not likely to fully explain the loss of bone mass. It is obvious that bone mass could also be regulated by muscle-derived biochemical factors such as myokines (160) (Fig. 4).

Studies in mice show that inhibition of the myokine myostatin pathway leads to an increase in bone mass, whereas (161) myostatin reduces osteoclast formation and bone destruction in a TNF-α transgenic mouse model of rheumatoid arthritis (162). Thus, whereas myostatin is a negative regulator of bone, it is a positive regulator of bone resorption.

Overexpression of IL-6 in IL-6 transgenic mice resulted in increased osteoclastogenesis (163). IL-6 appears to induce bone resorption through receptor activator of nuclear factor kappa-Β ligand (RANKL) -dependent enhanced osteoclastogenesis/osteoclast differentiation (164, 165) as well as via osteoblast-derived prostaglandin E2 (PGE2)-dependent osteoclast activation (166-168).

Given that trained people have low circulating basal levels of IL-6, whereas IL-6 increases with each bout of exercise, the interpretation of the findings above only makes sense if it is the chronic basal levels of IL-6 that modulate bone, rather than the acute peaks in IL-6 levels as also pointed out by Banfi (169).

Insulin-like growth factor 1 (IGF-1) has been shown to have a positive effect on bone formation (170). Muscle-derived IGF-1 can act on local osteoblasts that express the IGF-1 receptor and thereby promote bone formation (171).

Osteoglycin is a myokine (172) that appears to inhibit myoblast migration during myogenesis (173). Other myokines have been shown to affect bone metabolism, either positively (IGF-I, FGF-2, IL-15), or negatively (eg, TGF-β) (12, 174).

## Muscle–Liver Crosstalk

In order to maintain glucose homeostasis during exercise, glucose uptake in muscle is accompanied by increased glucose production from the liver (175). Mediators of endogenous glucose production include an increase in the portal venous glucagon-to-insulin ratio, epinephrine, and norepinephrine, but these factors cannot alone account for the rapid increase in glucose production (reviewed in, eg, (176)). In 1961, Goldstein (3) suggested that muscle cells might be able to produce a “humoral” component that could contribute to hepatic glucose production.

We infused rhIL-6 into resting human subjects and showed that acute administration of physiological concentrations of rhIL-6 did not influence whole-body glucose disposal, glucose uptake, or endogenous glucose production (177). However, in 2004, we published a study showing that during bicycle exercise IL-6 contributes to the increase in endogenous glucose production. Healthy young males underwent 2 hours of bicycle exercise on 3 separate occasions at (1) a relatively high intensity; (2) a low intensity, and (3) a low intensity + infusion of IL-6 to mimic the plasma levels of IL-6 observed during high-intensity exercise. The study showed the existence of direct muscle–liver crosstalk. Muscle-derived IL-6 plays a role in triggering glucose output from the liver during exercise in humans (176).

A murine study from 2018 showed that IL-6 treatment enhances AKT signaling and reduces gluconeogenic gene expression in livers from low and high fat fed mice, demonstrating that the beneficial effects of IL-6 on glucose and insulin homeostasis, in vivo, are maintained in obesity (178).

## Muscle–Gut Crosstalk

**Figure 5.**
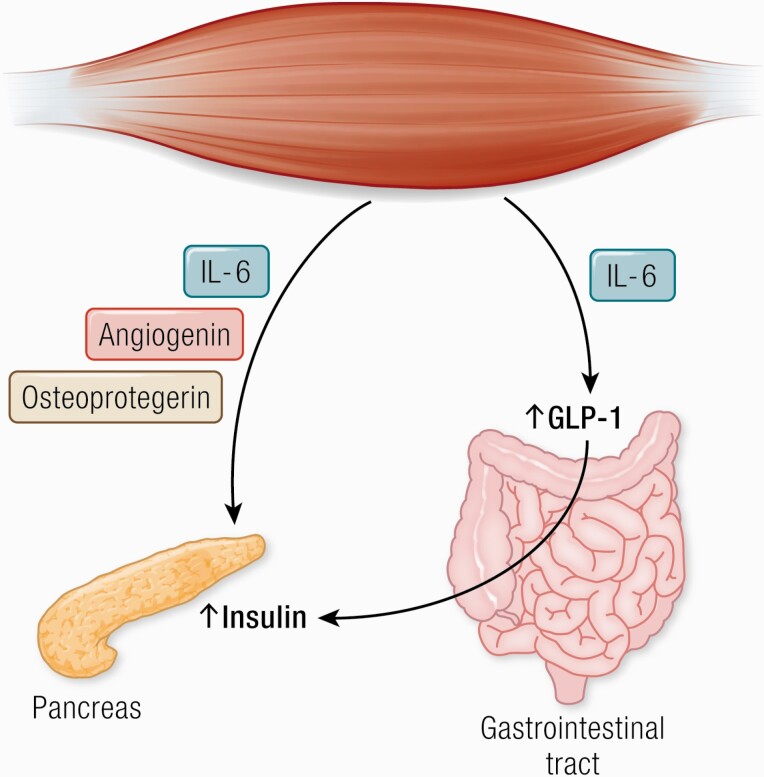
Angiogenin, osteoprotegerin, and IL-6 possess pancreatic β-cell protective actions against proinflammatory cytokines. IL-6 increases insulin secretion by inducing the expression of GLP-1 by the L cells of the intestine. Abbreviations: GLP-1, glucagon-like peptide 1.

A classic study by Ellingsgaard et al. (108) elegantly showed that acute elevations in IL-6 stimulates GLP-1 secretion from both intestinal L‐cells and pancreatic β‐cells, leading to improved secretion of insulin. This finding implicates IL‐6 in a beneficial regulation of insulin secretion and suggests that IL‐6 is involved in an endocrine loop that may protect against impaired glucose homeostasis (Fig. 5).

A recent study from our group (111) looked at the effects of IL-6 on postprandial glycemia and insulin secretion in humans and found that IL-6 delays the rate of gastric emptying, which is the most significant regulator of postprandial glucose (179). The study identifies a new role of human IL-6 being involved in gastric emptying and sparing insulin in a postprandial situation.

## Muscle–β-Cell Crosstalk

It is well established that exercise can enhance insulin sensitivity, whereas it is less clear whether exercise can improve insulin secretion and whether a communication exists between insulin-resistant skeletal muscle and pancreatic β-cells.

It has previously been shown that excessive concentrations of TNF-α induce insulin resistance in humans in vivo (180). We used TNF-α to induce insulin resistance in human myotubes. Conditioned media from muscle cells incubate with and without TNF-α were added to human and rat primary β-cells. The study identified a link between skeletal muscle and β-cells that is influenced by the insulin resistant state of the muscle (181).

Studying primary muscle cell cultures established from triceps brachii, soleus, and quadriceps led to the identification of angiogenin and osteoprotegerin, which were shown to be triceps-specific myokines that could mediate anti-inflammatory actions and protect β-cell survival (182). These results indicate that type I and type II muscles impact insulin secretion differentially in type 2 diabetes via specific myokines secretion.

Whereas TNF-α may inhibit β‐cell function indirectly, IL‐1β has been identified as a direct key player in β‐cell damage (183-189), although IL-1β inhibition with canakinumab did not reduce the incidence of diabetes (190). It has clearly been shown that IL‐6 positively regulates β‐cell mass in vivo by stimulating β‐cell proliferation and preventing apoptosis induced by metabolic stress (191). Therefore, exercise‐induced increase in IL‐6 production may be involved in protecting pancreatic β‐cell mass and function.

## Muscle–Vascular Bed Crosstalk

By stimulating the in vivo growth of functional type II muscle fibers, the Walsh group identified novel muscle-secreted factors (192). Follistatin-like 1 (FSTL1) was shown to be produced by both skeletal and cardiac muscle cells and is also termed a cardiokine (193).

FSTL1 has been shown to possess cardioprotective effects, promoting endothelial cell function and thereby revascularization in animal models of cardiac injury through a mechanism that includes nitric oxide synthase (194, 195). Circulating levels of FSTL1 may work as a biomarker as high concentrations of FSTL1 are seen in patients with systolic and diastolic heart failure (196, 197), and as FSTL1 levels exhibit prognostic significance in the acute coronary syndrome (198). Using a dog model, it was recently shown that FSTL1 can positively regulate myocardial substrate metabolism, in vivo (199).

## Muscle–Skin Crosstalk

Aging is associated with numerous alterations, including changes of the skin. Tarnopolsky and colleagues (200) demonstrated that endurance exercise improves age-associated skin changes in both mice and humans. They showed that exercise regulates muscular IL-15 expression via skeletal muscle AMPK. Elimination of muscle AMPK led to a weakening of skin structure, whereas IL-15 injections mimic some of the anti-aging effects of exercise on murine skin. The study supports the idea that exercise retards skin aging via a mechanism that involves muscle-derived IL-15.

## Muscle–Immune Inflammation Crosstalk

**Figure 6.**
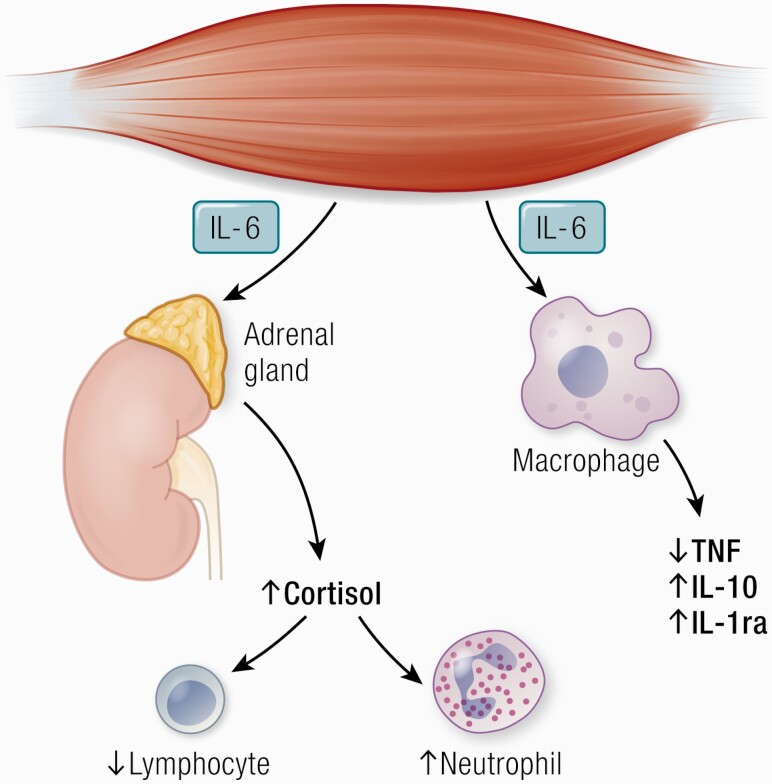
IL-6 has anti-inflammatory effects as it inhibits TNF production and stimulates the production of IL-1ra and IL-10. IL-6 stimulates cortisol production and thereby induces neutrocytosis and lymphopenia. Abbreviations: IL-1ra, IL-1 receptor antagonist; TNF, tumor necrosis factor.

During exercise, muscle works as an immunoregulatory organ with impact on leukocyte subset trafficking and inflammation (201) (Fig. 6).

### Lymphocyte and neutrophil trafficking

During exercise, lymphocytes and neutrophils are mobilized to the blood. Following long-duration exercise of high intensity, the concentration of lymphocytes falls below pre-exercise values whereas the neutrophil number continues to increase (202, 203). The acute exercise effect on lymphocytes and neutrophils is mediated by adrenaline, but the post-exercise reduction in lymphocyte number and the continuous increase in neutrophil number are mediated by both adrenaline and cortisol.

There are some indications that the exercise-induced rise in cortisol is mediated by IL-6. The infusion of IL-6 to mimic the effects of exercise led to an increase in cortisol and, consequently, a decrease in the lymphocyte number accompanied by an increase in neutrophil number (112).

Two other studies payed some support to a possible link between IL-6, lymphocyte number and cortisol. Carbohydrate ingestion during exercise blunted the exercise-induced IL-6 response, the increase in lymphocyte number as well as the cortisol (204, 205). Moreover, antioxidant supplementation totally inhibited the release of IL-6 from exercising human muscle as well as the exercise-induced increase in systemic levels of cortisol (206).

### The anti-inflammatory effects of exercise

Physical inactivity is associated with low-grade chronic inflammation, not least when a physical inactive lifestyle is associated with obesity (29, 142, 144, 145, 207-210).

In humans, exercise training can induce anti-inflammatory effects both acutely with each bout of exercise and via long-term training adaptation including reduction in abdominal adiposity. The exercise-induced acute increase in IL‐6 stimulates an anti-inflammatory systemic environment. Thus, IL-6 promotes an increase in the production of the anti-inflammatory cytokines, IL‐1 receptor antagonist (IL‐1ra) and IL‐10 (112). IL‐1ra inhibits IL‐1β signal transduction (211) and IL‐10 inhibits synthesis of TNF‐α (212).

We infused a very low dose of *Escherichia coli* endotoxin to healthy subjects, who were randomized to either rest or exercise prior to the endotoxin administration (113). Exercise prior to endotoxin totally blunted the increase in circulating levels of TNF‐α that was observed during a resting situation.

Previous studies in cultured human monocytes have shown that IL‐6 prevents endotoxin-induced TNF‐α production (213). Moreover, it was shown that IL‐6‐deficient knockout mice have elevated levels of TNF‐α (214). It was therefore expected that an infusion of rhIL-6 prior to endotoxin administration would also blunt the TNF‐α response in humans and this was in fact what was found (113).

Together these data show that an acute bout of exercise induces anti‐inflammatory effects that may at least partially be mediated by IL‐6, not excluding other anti-inflammatory factors such as adrenaline and cortisol, as previously discussed (142).

A recent murine study suggested that IL-6 may induce either pro- or anti-inflammatory actions depending on cell source (215). Using a mouse model with conditional expression of the *Il6* gene, it was found that IL6 derived from adipocytes increased, while IL6 derived from myeloid cells and muscle suppressed, macrophage infiltration of adipose tissue. The finding of opposite actions of IL-6, depending of the cell source, appeared to be due to a switch of IL6 signaling from a canonical mode (myeloid cells) to a noncanonical trans-signaling mode (adipocytes and muscle) which involved increased expression of the ADAM10/17 metalloprotease that enhances trans-signaling via the soluble IL6 receptor α (215).

Long-term anti-inflammatory effects are facilitated via exercise-training reduction in abdominal fat (216). In fact, an association has been established between physical inactivity and visceral fat in both rodents (217) and humans (133, 218-220). Accumulation of visceral fat, which is more inflamed than subcutaneous fat, leads to chronic systemic inflammation that predisposes to atherosclerosis, elevated blood lipids, insulin resistance, neurodegeneration, muscle waste, and anemia, factors that are likely to lead to decreased physical activity. Lack of exercise provokes accumulation of more visceral fat and thereby further enhances inflammation and hence a network of chronic diseases. Thereby, a vicious circle of chronic inflammation is established (29).

Exercise training will lead to a decrease in visceral and cardiac fat mass (136, 138, 221) and hence a decrease in circulating inflammatory molecules via a mechanism that involves exercise-induced increase in IL-6 (136), as described above.

## Muscle–Cancer Crosstalk

Epidemiological studies suggest that physical activity in leisure time reduces the risk of at least 13 different cancer types (41, 42, 222, 223). People who are physically active after a diagnosis of prostate cancer, colorectal cancer, and breast cancer have a higher survival rate than physically inactive people suffering from the same cancer types (121).

It is obvious, that many cancers are accompanied by systemic low-grade chronic inflammation and that such inflammation may drive tumor progression. Therefore, the anti-inflammatory effects of physical training may mediate some of the protective effects of exercise on cancer development (41).

Pernille Hojman and her team explored the effect of exercise on tumor growth in preclinical models (42, 222). She first established a B16F10 melanoma model and randomized tumor-bearing mice to voluntary wheel running or control. Running mice demonstrated a marked reduction in tumor volume and incidence across 6 different tumor models. The effects of exercise on cancer growth were mediated via a direct regulation of natural killer cells by a mechanism that involved epinephrine-dependent mobilization of natural killer cells to the circulation and an IL-6-dependent redistribution to tumors. Blocking IL-6 signaling during exercise abolished the exercise-induced inhibition of tumor growth. The findings in mice indicate that IL-6 may have a role in mediating anti-cancer effects.

A few mechanistic studies have demonstrated a potential role of other myokines, including Oncostatin M, irisin, and SPARC in the suppression of breast and colon cancer growth (41, 224-227).

## Myokines and Other “Kines”: Adipokines, Hepatokines, and Batokines

The views on organ crosstalk in health and disease have changed over the past 30 years.

It all began with the work from the Spiegelman and Flier laboratories in 1987 (228) that defined adipose tissue as an endocrine organ by the identification of a secretory protein, called adipsin. This was followed by a landmark finding by Friedman and his team (229), who identified leptin. Since then the list of adipokines have included, for example, adiponectin, resistin, and visfatin (230, 231).

**Figure 7.**
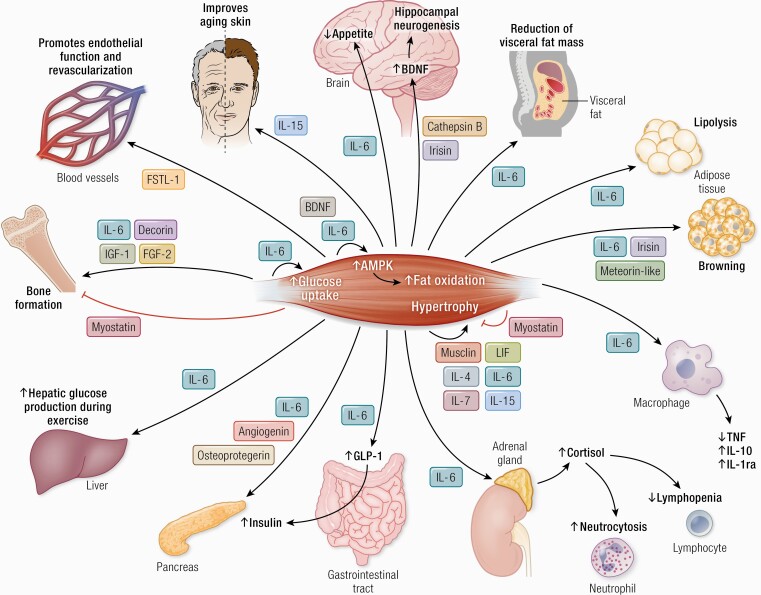
Cathepsin B and irisin cross the blood–brain barrier and stimulate BDNF production and hippocampal neurogenesis. IL-6 stimulates appetite and lipolysis and decreases visceral fat mass. Irisin, meteorin-like, and IL-6 have a role in “browning” of white adipose tissue. IL-15 improves aging skin. Decorin, IL-6, IGF-1 and FGF-2 positively regulate bone formation. Myostatin negatively regulate bone formation. Musclin, LIF, IL-4, IL-6, IL-7, and IL-15 promote muscle hypertrophy. Myostatin inhibits muscle hypertrophy. BDNF and IL-6 are involved in AMPK-mediated fat oxidation. IL-6 enhances insulin-stimulated glucose uptake and stimulates glucose output from the liver, but only during exercise. IL-6 increases insulin secretion by inducing the expression of GLP-1 by the L cells of the intestine. IL-6 has anti-inflammatory effects as it inhibits TNF production and stimulates the production of IL-1ra and IL-10. IL-6 stimulates cortisol production and thereby induces neutrocytosis and lymphopenia. FSTL-1 improves endothelial function and revascularization of ischemic blood vessels. Angiogenin, osteoprotegerin and IL-6 possess pancreatic β-cell protective actions against proinflammatory cytokines. Abbreviations: AMPK, 5′-AMP-activated protein kinase; BDNF, brain-derived neurotrophic factor; FGF-2, fibroblast growth factor 2; FGF-21, fibroblast growth factor 21; FSTL-1, follistatin-related protein 1; GLP-1, glucagon-like peptide 1; IGF-1, insulin-like growth factor I; IL-1ra, IL-1 receptor antagonist; LIF, leukemia inhibitory factor; TGF-β, transforming growth factor β; TNF, tumor necrosis factor.

The identification of muscle as a secretory organ began with the finding of muscle-derived IL-6 in 2000 (5) and the subsequent definition of myokines in 2003 (4), and led via the work of many research groups later to the identification of hundreds of myokines. The present review identifies crosstalk between muscle and several other organs, including brain, adipose tissue, bone, liver, gut, pancreas, vascular bed, and skin. Moreover, several myokines signal within the muscle itself (Fig. 7).

Recently, a novel group of liver-derived exercise factors has been identified. Hepatokines include FGF-21, follistatin, angiopoietin-like protein 4, heat shock protein 72, and IGF binding protein, which are all released from the liver during or immediately after an exercise bout (232). These hepatokines increase in the circulation after muscle work and appear to be involved in mediating some of the metabolic effects of exercise.

The latest news regarding other “kines” started with the identification of classic brown adipose tissue in adult humans (233), which led to the batokine concept. Most recently, 101 proteins were exclusively quantified into brown and not white adipocyte tissue by proteomic-based identification (234).

However, among the “kines,” focus is still primarily on myokines and hepatokines when it comes to mediating exercise-induced communication between muscle and other organs. Lack of physical activity is associated with a large network of diseases, including type 2 diabetes, cardiovascular diseases, cancer, dementia, and osteoporosis (72, 121), and it is likely that the detrimental effects of lack of exercise to some degree is mediated by a lack of myokine release and/or resistance to the effects of myokines. The identification of new myokines and their specific roles will likely lead to novel therapeutic targets for lifestyle-related diseases. However, the biological identification of several myokines has turned these molecules into useful biomarkers for monitoring the amount, intensity, and mode of exercise that is sufficient to induce specific physiological and metabolic responses for people with, for example, cancer, diabetes, or neurodegenerative diseases.

## Abbreviations

AMPK: adenosine 5′-monophosphate-activated protein kinase
BDNF: brain-derived neurotrophic factor
CTSB: cathepsin B
FGF: Fibroblast growth factor
FSTL1: follistatin-like 1
GLP: glucagon-like peptide
IGF-1: insulin-like growth factor 1
IL: interleukin
LIF: leukemia inhibitory factor
Metrnl: meteorin-like
mRNA: messenger ribonucleic acid
TGF-β: transforming growth factor β
TNF: tumor necrosis factor
UCP1: uncoupling protein 1

## Additional Information

***Disclosure Summary:*** We have nothing to disclose.

***Data Availability:*** Data sharing is not applicable to this article as no datasets were generated or analyzed during the current study.
