# Corrigendum to: “Muscle-Organ Crosstalk: The Emerging Roles of Myokines”

**DOI:** 10.1210/endrev/bnaa024

**Published:** 2020-11-05

In the above-named article by Severinsen MCK and Pedersen BK (*Endocrine Reviews*. 2020;41(4):594–609; doi: 10.1210/endrev/bnaa016), the following error occurred in the published paper: “In Figure 2, Figure 7, and the graphical abstract, an arrow shows that IL-6 stimulates appetite. The opposite is true: IL-6 inhibits appetite, as is stated in the text.”

In the Graphical Abstract, the upward-pointing arrow next to the word “appetite” has been changed to a downward-pointing arrow online.

**Graphical Abstract:**
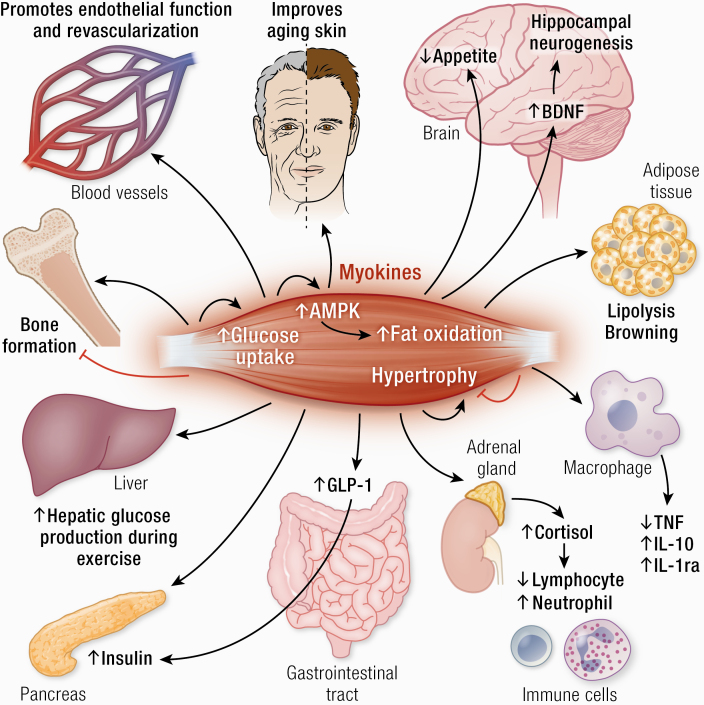


The legend to Figure 2 stated “IL-6 stimulates appetite” and has been changed online to “IL-6 inhibits appetite”. The upward-pointing arrow indicating increased appetite on the figure has been changed online to a downward-pointing arrow.

**Figure 2.**
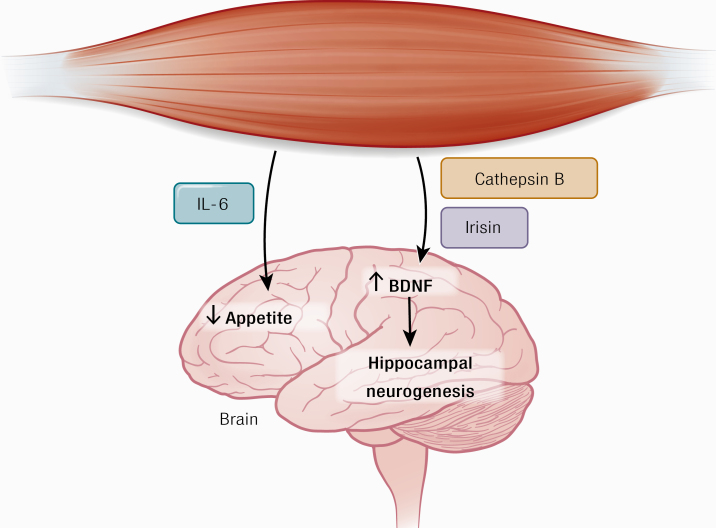
Cathepsin B and irisin cross the blood–brain barrier and stimulate BDNF production, which leads to hippocampal neurogenesis. IL-6 inhibits appetite. Abbreviations: BDNF, brain-derived neurotrophic factor.

The legend to Figure 7 stated “IL-6 stimulates appetite and lipolysis and decreases visceral fat mass” and has been changed online to “IL-6 inhibits appetite, stimulates lipolysis and decreases visceral fat mass.” The upward-pointing arrow indicating increased appetite on the figure has been changed online to a downward-pointing arrow.

**Figure 7.**
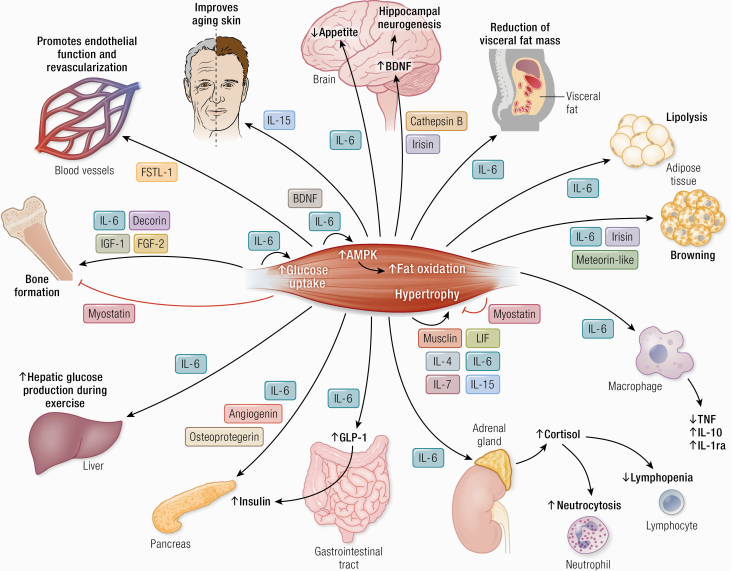
Cathepsin B and irisin cross the blood–brain barrier and stimulate BDNF production and hippocampal neurogenesis. IL-6 inhibits appetite and lipolysis and decreases visceral fat mass. Irisin, meteorin-like, and IL-6 have a role in “browning” of white adipose tissue. IL-15 improves aging skin. Decorin, IL-6, IGF-1 and FGF-2 positively regulate bone formation. Myostatin negatively regulate bone formation. Musclin, LIF, IL-4, IL-6, IL-7, and IL-15 promote muscle hypertrophy. Myostatin inhibits muscle hypertrophy. BDNF and IL-6 are involved in AMPK-mediated fat oxidation. IL-6 enhances insulin-stimulated glucose uptake and stimulates glucose output from the liver, but only during exercise. IL-6 increases insulin secretion by inducing the expression of GLP-1 by the L cells of the intestine. IL-6 has anti-inflammatory effects as it inhibits TNF production and stimulates the production of IL-1ra and IL-10. IL-6 stimulates cortisol production and thereby induces neutrocytosis and lymphopenia. FSTL-1 improves endothelial function and revascularization of ischemic blood vessels. Angiogenin, osteoprotegerin and IL-6 possess pancreatic β-cell protective actions against proinflammatory cytokines. Abbreviations: AMPK, 5′-AMP-activated protein kinase; BDNF, brain-derived neurotrophic factor; FGF-2, fibroblast growth factor 2; FGF-21, fibroblast growth factor 21; FSTL-1, follistatin-related protein 1; GLP-1, glucagon-like peptide 1; IGF-1, insulin-like growth factor I; IL-1ra, IL-1 receptor antagonist; LIF, leukemia inhibitory factor; TGF-β, transforming growth factor β; TNF, tumor necrosis factor.

Doi: 10.1210/endrev/bnaa016

